# International Microsurgery Club and World Society for Reconstructive Microsurgery Webinar: Career Building in Microsurgery

**DOI:** 10.1055/a-2229-3420

**Published:** 2024-04-08

**Authors:** Joachim N. Meuli, Jung-Ju Huang, Susana Heredero, Wei F. Chen, Tommy NJ Chang

**Affiliations:** 1 Department of Plastic and Hand Surgery, Lausanne University Hospital, Lausanne, Switzerland; 2 Department of Plastic and Reconstructive Surgery, Chang-Gung Memorial Hospital, Taoyuan, Taiwan; 3 UGC de Cirugía Maxilofacial, Hospital Universitario Reina Sofía, Córdoba, Spain; 4 Department of Plastic Surgery, Cleveland Clinic, Cleveland, Ohio

**Keywords:** medical education, microsurgery, surgical training

## Abstract

Career building can be challenging for young surgeons, especially when topics such as lifestyle, work–life balance and subspecialization arise. Suggestions and advice from senior colleagues is very valuable but many young surgeons do not have such opportunities or are limited to a few senior surgeons. The International Microsurgery Club (IMC), in collaboration with the World Society of Reconstructive Microsurgery, organized a combined webinar for this topic and invited world renowned microsurgery masters polled by the IMC members to join, including Prof. Peter Neligan (Emeritus from University of Washington, United States), Prof. Raja Sabapathy (Ganga Hospital, India), Dr. Gregory Buncke (The Buncke Clinic, United States), Prof. Isao Koshima (Hiroshima University Hospital, Japan), Prof. David Chwei-Chin Chuang (Chang Gung Memorial Hospital, Taiwan), and Prof. Eric Santamaria (Hospital General Dr. Manuel Gea Gonzalez, Mexico) on May 1, 2022. Prof. Joon-Pio Hong (Asan Medical Center, South Korea) and Prof. Fu-Chan Wei (Chang Gung Memorial Hospital, Taiwan) were also selected but unfortunately could not make it and were therefore invited to another event in April 2023, summarized in a recently published paper.

There is ample literature reporting on different aspects of developing a microsurgical career but the goal of this session was to offer an opportunity for direct exchange with experienced mentors. Moreover, insights from experienced microsurgeons from different part of the world were more likely to offer different perspectives on aspects such as career building, failure management, and team culture. This webinar event was moderated by Dr. Jung-Ju Huang (Taiwan), Dr. Susana Heredero (Spain), and Dr. Wei F. Chen (United States).

This paper summarizes the lessons from the “Career Building in Microsurgery” webinar ( Fig. 1 ) and completes the paper recapitulating the “Dialogue with the Giants of Microsurgery” webinar published in 2023. 1 These webinars aim at offering a direct exchange with mentors. completing the existing literature focused on factors impacting the choice of a microsurgical career. 2 3 4 . The introduction was an open discussion about change, innovation, and dogma. Prof. Chuang mentioned his belief that microsurgeons (and people, in general) can be split into two different groups: those who follow routine processes (group A) and those who pursue a constant quest for changes (group B). In this second group, change often starts simply with interest. Interest induces passion and passion helps to discover or select potential changes. Prof. Chuang strongly encouraged microsurgeons in training to ask themselves how willing they are to follow their passion, including when this implies to challenge current dogma and to appear as an “original.” Prof. Neligan, Prof. Buncke, and Prof. Sabapathy suggested to always try to innovate based on what one already knows, to focus on patient-based problems and to always publish the results, no matter whether positive or negative. In their opinion, this does not only improve the quality of scientific publishing in reconstructive microsurgery but also increases trust in authors. Lastly, Prof. Santamaria highlighted the importance for microsurgeons in training to build their own stairs, one step after another, to improve. Jumping steps is no recipe for a successful career.

**Fig. 1:**
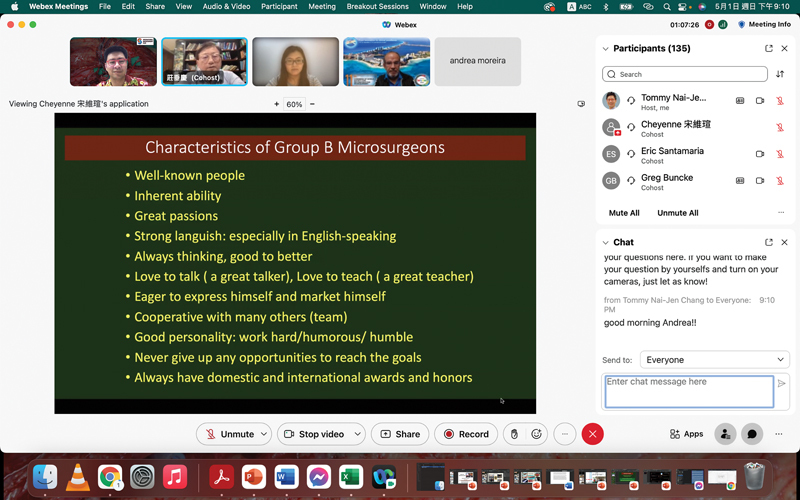
IMC webinar: career building in microsurgery. IMC, International Microsurgery Club.

## Q: How do you Know you are Ready or Good Enough to Innovate?

According to Prof. Santamaria and Prof. Neligan, there is no clear point where one suddenly becomes “ready to innovate.” It is rather a continuous process of challenging things from the very beginning. Prof. Sabapathy shared a strategy to evaluate when one is ready to innovate: he suggested that microsurgeons in training always outline a clear plan when they assist in a procedure. If the surgeon in charge does not follow this plan or does not make the expected decision, the surgeon in training should ask why. If, at one point, no satisfying answer can be brought, there is room for innovation. Prof. Koshima mentioned that in his opinion, it is essential to maintain fundamental research activities in parallel to clinical training to innovate. He suggested to attend research meetings related to other specialties and departments as this gives exposure to new fields and ideas.

Lastly, Prof. Neligan insisted on the superiority bias: statistically, most people are average and this rule applies to microsurgeons. Despite this, people tend to think that they are better than average. It is, therefore, crucial to receive feedback about your abilities during your training and afterwards. Prof. Buncke mentioned this as well, highlighting the fact that communication is key when advancing through a career.

## Q: What is the Main Cause for the Lack of Microsurgeons?

Prof. Sabapathy believed we do not have enough microsurgeons because we do not manage to attract medical graduates into this field. He highlighted the importance of showing that one can have a great quality of life while being a microsurgeon. Prof. Santamaria and Prof. Neligan added that the compensation mechanisms in place often make reconstructive microsurgery an unattractive activity and surgeons tend to switch to aesthetic procedures. However, lack of support from hospital management, lack of patients referral, and general obstacles to the development of practices also contribute to microsurgeons switching to another field of surgery to sustain a living.

## Q: Where do you Get your Ideas from?

Prof. Sabapathy believed that people need time for themselves to develop new ideas and surgeons tend to have too little time dedicated to themselves. For him, long flights were and still are fantastic occasions to have undisturbed time. Prof. Buncke suggested that whenever one feels like he or she reached a plateau, going to a meeting or conference is a great way to get new stimulations and ideas as well. Prof. Chuang's opinion is that having new ideas is not an accidental process. New ideas require solid foundations and extensive theoretical knowledge provides more opportunity for new ideas to emerge.

## Q: What do you Think is the Greatest Unsolved Problem in Microsurgery Right Now?

Prof. Neligan suggested that there might be several “greatest problems,” depending on the subfield one is working in. One of the most exciting thing in his opinion, shared by Prof. Sabapathy, is the change from standard microscope to 3D video as well as the introduction of robotic microsurgery. There is a natural limit to eye–hand coordination and robots can assist when this normal tremor becomes limiting. Prof. Santamaria shared his fascination with bioengineering and the concept of regenerating body part which would revolutionize reconstructive surgery. Another great unsolved problem shared by Prof. Buncke is the lack of awareness of the general public and medical community about microsurgery.

## Q: How do you Keep your Passion for Microsurgery Alive?

Prof. Chuang believed that given enough passion about microsurgery, one can continue forever. Prof. Sabapathy and Prof. Santamaria shared this positive philosophy, reminding the audience that the work–life balance of many other professions is challenging as well, but added that having a great team is essential as people push each other to constantly improve. Prof. Neligan and Prof. Buncke insisted on how fun microsurgery can be, no matter at what time. One last strategy, shared by many of the panelists, was to maintain physical activity besides work.

## Q: How you do Deal with the Bullying from Seniors ?

Prof. Koshima remarked that pioneers are often bullied because they challenge their seniors, both in professional and private aspects of life. His advice is to find support among peers around the world (as in the International Microsurgery Club [IMC]). Pioneers tend to even get excluded from their own specialty societies because these societies are not ready to accommodate challengers. Prof. Neligan agreed, mentioning that one of the practical ways of dealing with this is to bypass the hierarchy, using platforms such as IMC to contact experts in other institutions who can provide support. A general side remark was that the people bullying are not always excessively bad people. Sometimes, they are just people with strong confidence in their own beliefs and reluctant to have those beliefs challenged. Prof. Neligan highlighted that jealousy sometimes plays a big role as well in such situations.

## Q: How do you Deal with Repeated Failure in Surgery?

Prof. Neligan, Prof. Santamaria, and Prof. Sabapathy insisted on the fact that understanding why failure occurred is the key. To avoid repeating it, one has to do something differently. Always having a lifeboat procedure planned is as important as realizing when one has to give up. Sometimes, what a surgeon really needs is a bit of sleep and advice from colleagues.

## Q: How to Maintain a Work–Life Balance?

Prof. Buncke acknowledged that this is a very difficult problem but it is not specific to microsurgery. The key, in his opinion, is making sure your family understands that you care about them. The “work hard, play hard” mentality might help as well by making clear that the time dedicated to them is only dedicated to them. Prof. Sabapathy received a similar advice early on to always be there for his family when there is an issue. In his perspective, he makes little effort at work because this job is fun for him. He, however, makes great efforts for his family afterwards. Lastly, Prof. Neligan shared his personal attempt at always having dinner with his family throughout his career, with sometimes moderate success.

## Q: When is the Best Time to Retire from Microsurgery?

Prof. Neligan suggested to retire when going to work is no longer fun. For him, the COVID-19 pandemic was the trigger and he now enjoys his retirement while remaining engaged in textbooks and meetings. The exact answer to this question might be different for everybody but the only risk is to stay for too long. Prof. Sabapathy's perspective is that retirement becomes less an issue if one remains available for his or her colleagues should they need advice in the future. In his perspective, you do not grow old but if you stop growing, you become old.

## Conclusion

Gathering masters to share their experience using a webinar format is an efficient and effective way for brainstorming. Everyone can access it, just connect successfully, and interact with the masters without any burden. If the members missed it or want to review it, they can also check our customized website (International Microsurgery Website) for the record at: https://imw.global/imw/#/video/category/5e9486e80cb9c000015854f3/62845061eecc2c000102ab3b .
